# Conserved and Divergent Phytochemical Profiles in Native and Micropropagated *Micromeria croatica* (Pers.) Schott: An LC-HRMS Study Across Solvent Extracts

**DOI:** 10.3390/plants14192971

**Published:** 2025-09-25

**Authors:** Svetlana M. Tošić, Marija Ilić, Ljubica Svilar, Jelena Nikolić, Milan Mitić, Violeta Mitić, Vesna P. Stankov Jovanović

**Affiliations:** 1 Department of Biology and Ecology, Faculty of Sciences and Mathematics, University of Niš, Višegradska 33, 18000 Niš, Serbia; svetlana.tosic@pmf.edu.rs; 2 Veterinary Specialized Institute Niš, Dimitrija Tucovića br. 175, 18106 Niš, Serbia; marija.fertico@gmail.com; 3 Centre Cardiovasculaire et Nutrition (C2VN), CRIBIOM, Aix Marseille Université, 13007 Marseille, France; ljubica.svilar@univ-amu.fr; 4 Department of Chemistry, Faculty of Sciences and Mathematics, University of Niš, Višegradska 33, 18000 Niš, Serbia; jelena.cvetkovic@pmf.edu.rs (J.N.); milan.mitic1@pmf.edu.rs (M.M.); violeta.mitic@pmf.edu.rs (V.M.)

**Keywords:** *Micromeria croatica*, Balkan endemic plants, micropropagation, *in vitro* culture, LC-HRMS metabolite profiling, phytochemical profiling, methanol, ethyl acetate, hexane extracts, plant secondary metabolites

## Abstract

*Micromeria croatica* (Pers.) Schott is a Balkan endemic of the Lamiaceae family, valued for its aromatic and medicinal properties, but it is threatened by its limited natural distribution. Micropropagation offers a sustainable method for securing biomass and provides material for chemical studies. In this work, we present the first LC-HRMS profiling of extracts (in methanol, ethyl acetate, and hexane) obtained from both native and micropropagated plants. A total of 29 metabolites were identified. A diverse spectrum of secondary metabolites was identified, including phenolic acids (gallic acid monohydrate, vanillic acid, trans-cinnamic acid), flavonoids (luteolin-7-O-rutinoside, diosmetin-7-O-glucoside, kaempferol-O-rutinoside, eriocitrin), and terpenoids (ursolic acid, tanshinone I, riligustilide). The analysis revealed that all compounds detected in native plants were also present in micropropagated material, demonstrating the preservation of the characteristic phytochemical profile in vitro. Moreover, several compounds, such as apigenin, apigenin-7-O-glucuronide, isomaltopaeoniflorin, and methoxylated flavones, were found exclusively in micropropagated samples, indicating that tissue culture may enhance the chemical diversity of the species. Ethyl acetate extracts showed the highest degree of overlap between native and in vitro plants, whereas methanol and hexane extracts contained a greater number of unique metabolites in micropropagated material. This first comprehensive phytochemical report on *M. croatica* highlights the importance of micropropagation as a sustainable strategy for conserving rare species while ensuring a reliable source of bioactive metabolites.

## 1. Introduction

The family Lamiaceae is one of the most species-rich plant families and is well known for its chemical diversity. Within this family, members of the tribe Mentheae have garnered attention due to their aromatic oils and secondary metabolites, many of which play a role in traditional medicine and modern phytotherapy. Phylogenetic work has shown that the diversity observed today in Mentheae is the result of complex biogeographic histories, particularly in the Mediterranean Basin, where repeated climatic fluctuations have shaped both distribution and diversification [1].

The genus *Micromeria* belongs to this tribe and includes many small aromatic perennials that occupy ecologically specialized habitats. Phytochemical studies conducted on various *Micromeria* species have revealed that their essential oils and phenolic profiles are both species-specific and highly variable, depending on geography and microenvironment [2]. Early chemical analyses of Balkan *Micromeria* species already pointed out a predominance of monoterpenes and sesquiterpenes, but also highlighted that considerable differences may occur between populations and even among individual plants [3].

It is now widely recognized that the environment exerts a strong influence on the chemical composition of Lamiaceae plants. Factors such as light intensity, soil type, and water availability are known to alter the yield and quality of essential oils [4]. Seasonal variation is another important determinant, often producing measurable differences in volatile profiles even within a single growing season [5]. Likewise, studies along altitudinal gradients have shown that elevation can affect both the total amount of oil produced and the proportions of individual compounds [6]. For a narrow endemic such as *Micromeria croatica*, which occurs across a broad elevational range but in very specific rocky niches, these observations raise the question of whether chemical variation in the wild reflects true genetic differences or mainly environmental plasticity.

This distinction is not only of botanical interest but also has practical importance. If the phytochemical profile of *M. croatica* is strongly shaped by environmental conditions, then any application of its extracts—for example, in food, cosmetic, or pharmaceutical products—will be subject to variability. On the other hand, if certain features of its chemistry remain constant regardless of growth conditions, then these traits can be considered stable markers, useful for quality control and for chemotaxonomic purposes. Advanced analytical techniques, such as liquid chromatography coupled with high-resolution mass spectrometry (LC-HRMS), now enable the detailed profiling of metabolites, capturing both phenolic acids, flavonoids, terpenoids, and other small molecules in a single run [7]. This approach has already been applied to other *Micromeria* species, revealing a wide array of bioactive compounds [8].

For species like *M. croatica*, which are endemic and naturally restricted, the challenge is that wild populations cannot supply the biomass needed for either large-scale analysis or any future applications. Excessive collection could pose a threat to already limited habitats. Micropropagation offers a solution here. By establishing in vitro culture protocols, it is possible to produce large numbers of uniform, pathogen-free plants under controlled conditions, thereby avoiding damage to natural populations. Tissue culture also provides an opportunity to study plant metabolism in a controlled environment, thereby reducing the “noise” introduced by fluctuating field conditions [9].

From a conservation perspective, micropropagation is increasingly regarded as a vital component of ex situ conservation strategies for endangered or endemic species. It has been successfully used for many medicinal and aromatic plants, allowing both the multiplication of planting stock and the long-term conservation of genetic resources [10]. In combination with bioreactor systems, micropropagation can be scaled up to produce thousands of plantlets in relatively little time, while keeping costs and contamination risks low [11].

The family Lamiaceae offers several instructive examples. In *Rosmarinus officinalis*, temporary immersion systems yielded vigorous plants whose essential oil content and composition were comparable to those of conventionally grown material [12]. In *Salvia apiana*, in-vitro propagation provided a reliable supply of material for chemical analyses and helped reduce pressure on wild populations that are under threat from overharvesting [13]. Micropropagation has also been successfully applied to *Origanum vulgare* and several *Thymus* species, generating uniform lines that retain their characteristic carvacrol-thymol chemotypes [14,15]. Particularly relevant for the Balkans is *Sideritis scardica*, another regional endemic, for which micropropagation has been developed as both a conservation tool and a means of ensuring reproducible phytochemical profiles [16].

These case studies illustrate two key points. First, micropropagation is not merely a conservation technique, but also a practical method for producing chemically consistent plant material for research and industry. Second, the comparison between native and micropropagated plants is essential, because while in many cases chemical profiles are preserved, in others the tissue culture environment may favor or suppress certain pathways. Plant hormones, light intensity, or carbon source in the culture medium can all steer metabolism in particular directions [17]. This dual role—stability in some respects, plasticity in others—means that careful chemical characterization of in vitro- derived plants is indispensable.

Another advantage of micropropagation is that it produces pathogen-free starting material, usually through meristem culture, often combined with thermotherapy. Systemic pathogens are known to alter secondary metabolism, and their elimination ensures that chemical differences are due to genetic or environmental factors rather than hidden infections [18]. For researchers interested in linking genotype, environment, and metabolite composition, this clean starting point is invaluable.

Considering the background outlined above, *Micromeria croatica* is an ideal subject for such a comparison. As an endemic species of the Balkan Peninsula, it carries both conservation value and pharmacological promise. Its essential oils and phenolics are reported to vary among populations, but it remains unclear how much of this variation is environmentally induced and how much is genetically fixed. By cultivating plants under in vitro conditions and comparing them with wild populations, we can assess whether the chemical signatures of this species remain stable (“indifference”) or shift significantly depending on the propagation method.

The genus *Micromeria* already boasts a remarkable array of bioactive compounds documented in various species. For instance, ethanolic extracts of *M. croatica*, along with *M. juliana* and *M. thymifolia*, show potent antioxidant effects—*M. Croatica* even outperformed the synthetic antioxidant BHT [19]. In *M. frivaldszkyana*, rosmarinic acid levels reach extraordinarily high concentrations (over 2040 mg per 100 g dry weight), and this species also demonstrates measurable antimicrobial activity—specifically against *Listeria monocytogenes*—in addition to strong activity in multiple antioxidant assays [20]. In another example, the essential oil of *M. myrtifolia* is rich in sesquiterpenes such as β-caryophyllene (40.8%) and α-copaene (17.9%), and has demonstrated not only antioxidant activity across various assays but also enzyme-inhibitory potency—particularly against tyrosinase and α-amylase—suggesting potential for food and cosmetic applications [21].

The present study provides the first comprehensive LC-HRMS profiling of *Micromeria croatica* extracts of different polarities (methanol, ethyl acetate, and hexane) obtained from both native and micropropagated plants. Our aims were twofold: to compare the metabolite composition between wild and in vitro–derived material, and to assess whether micropropagation can serve as a sustainable and reproducible source of phytochemically representative biomass.

## 2. Results

It is well established that both solvent polarity and extraction procedure strongly affect the yield and profile of bioactive metabolites [22]. Therefore, extracts were prepared using methanol, ethyl acetate, and hexane to recover compounds of varying polarities.

### 2.1. Liquid Chromatography Coupled to High Resolution Mass Spectrometry

**Figure 1:**
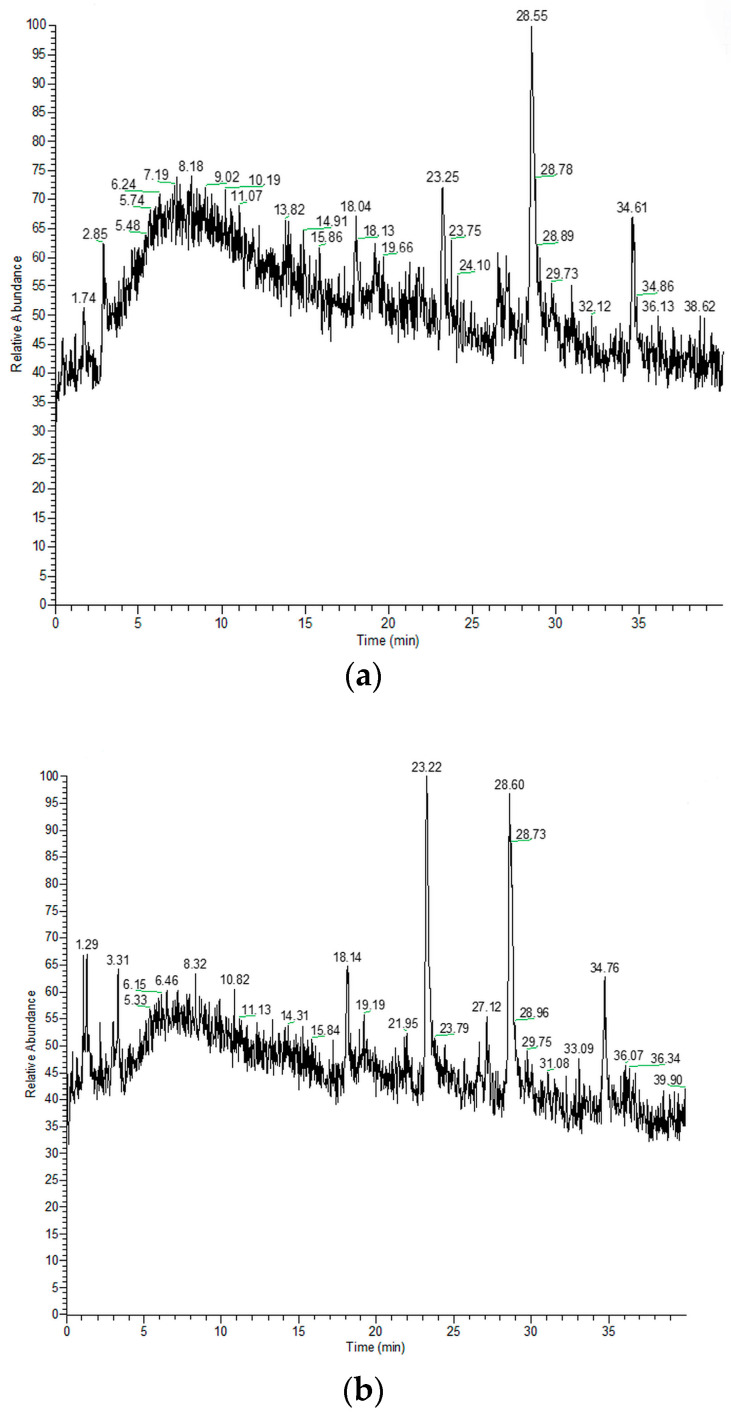
The total ion chromatograms of methanol extracts of native (**a**) and micropropagated (**b**) *M. croatica*.

**Figure 2:**
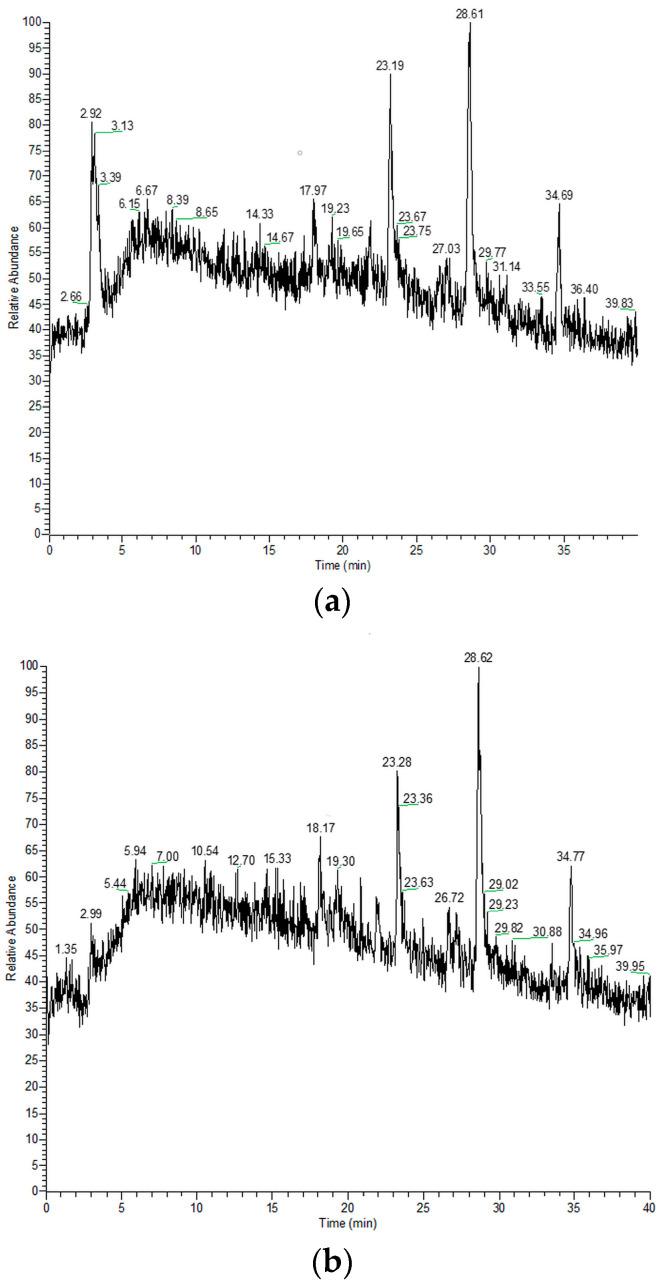
The total ion chromatograms of ethyl acetate extracts of native (**a**) and micropropagated (**b**) *M. croatica*.

**Figure 3:**
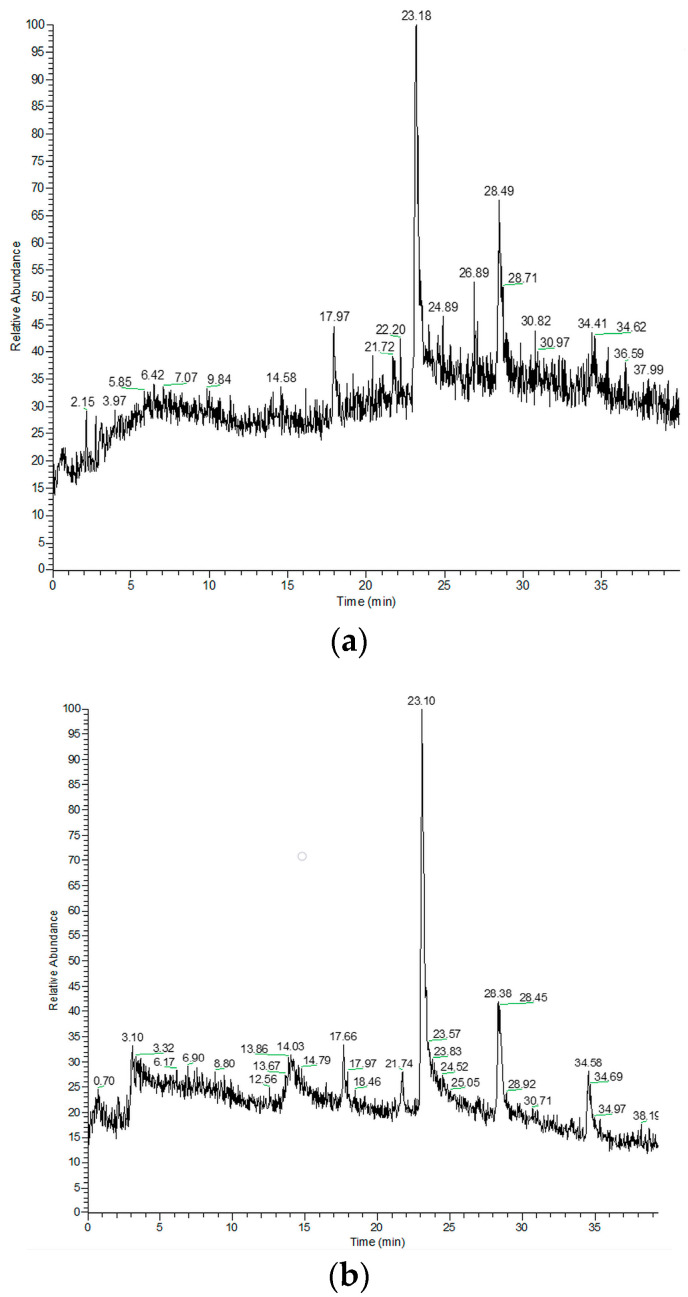
The total ion chromatograms of hexane extracts of native (**a**) and micropropagated (**b**) *M. croatica*.

Our study provides insight into the composition of different polarity constituents of *M. croatica*, both from natural sources and micropropagated. After preparing extracts, the following step was liquid chromatography. Total ion chromatograms (TIC) of methanol, ethyl acetate, and hexane extracts from native and micropropagated plants are presented in Figure 1, Figure 2 and Figure 3.

The TIC served as the starting point for generating corresponding mass spectra of plant extract constituents, based on their retention times and m/z ratios. Each of the mass spectra was then compared to the literature data, which were produced under the same ionization conditions, and in such a way, the chemical composition for each *M. croatica* (native and micropropagated) extract was determined. For each of the determined compounds, quantification was done and expressed as a percentage of the compound in the extract (Table 1, Table 2 and Table 3).

The determination of the compounds in all extracts revealed a total of 28 different compounds, present in the extracts. The results of LC-HRMS analysis are presented in Table 4.

**Figure 4:**
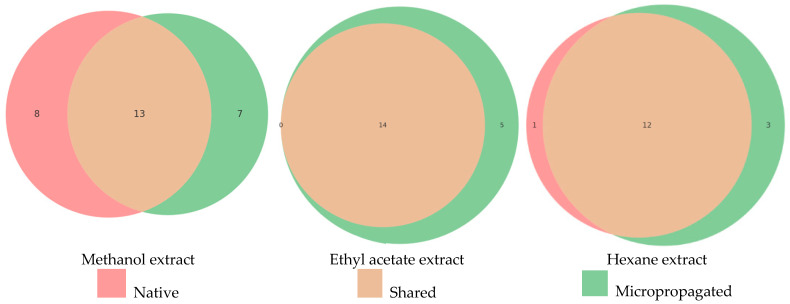
Venn diagrams for methanol, ethyl acetate and hexane extracts of native and micropropagated *M. Croatica* compounds.

Comparative LC-HRMS profiling of native and micropropagated plants identified a total of 28 unique compounds (Table 1, Table 2 and Table 3). The overlap and divergence in compound presence across solvents are visualized in Venn diagrams (Figure 4).

Overall, the methanol extracts showed the highest variability between wild and micropropagated plants, while ethyl acetate extracts indicated a trend toward reduced compound diversity in native plants.

### 2.2. Statistical Comparison of Native and Micropropagated M. croatica Extracts

Chi-square tests were performed to determine whether observed differences between native and micropropagated samples were statistically significant [37].

Methanol extracts: 28 compounds were detected (8 unique to native, 7 unique to micropropagated, and 13 shared). The χ^2^ test yielded 11.93175 with *p* = 0.99155, indicating no significant difference between the two profiles. LC-HRMS profiles of the methanol extract of the analyzed species showed that both plants, from native and micropropagated, share a substantial proportion of compounds (13 common). Micropropagation preserves most of the native phytochemicals, although some differences in minor constituents may occur.

Ethyl acetate extracts: Qualitative LC-HRMS analysis revealed both shared and unique compounds between the native and micropropagated *M. croatica* plants. A total of 19 compounds were detected in the micropropagated *M. croatica* extract, while 14 compounds were identified in the native extract, all of which were shared with the micropropagated extract. No single compound was detected in the native extract, which is different from the micropropagated.

The χ^2^ test yielded 6.389098 with *p* = 0.99683, suggesting a tendency toward divergence, although this difference was not statistically significant.

Hexane extracts: 13 compounds were identified in native and 15 in micropropagated plants, with χ^2^ = 3.876923 and *p* = 0.99811, confirming no significant difference. Of a total number of identified compounds, one was present solely in the extract of the native *M. Croatica*, 12 were shared, and 3 were found only in the micropropagated extract. The chi-square test showed no significant difference in the qualitative LC-HRMS profiles between the native and micropropagated *M. croatica*. This suggests that the overall presence/absence of secondary metabolites is largely conserved between naturally grown and in vitro propagated plants when extracted with hexane.

These results indicate that micropropagation largely preserves the qualitative phytochemical fingerprint of *M. croatica*. However, minor shifts occur, particularly in compounds of intermediate polarity.

## 3. Discussion

The methanol extracts of native and micropropagated *Micromeria croatica* revealed a partially overlapping but distinctly divergent phytochemical profile. A set of core metabolites was shared between both extracts, including albiflorin, trans-cinnamic acid, vanillic acid, gallic acid monohydrate, luteolin-7-O-rutinoside, kaempferol-O-rutinoside, diosmetin-7-O-glucoside, diosmetin-6-C-glucoside, kaempferol-O-rutinoside-3-O-rhamnoside, alisol F, apigenin, ursolic acid, tanshinone I, and riligustilide. The consistency of these compounds in both native and in vitro cultivated material suggests that they represent stable chemotaxonomic markers of the species [38].

Nevertheless, clear differences were evident between the two profiles. The native plants exhibited unique constituents absent in the micropropagated material, including dihydroxycholesterol, dihydroxymethoxyflavone-glucoside, cycloartenol trans-ferulate, salvinorin C, eriocitrin, and diosmetin-7-O-glucoside. These metabolites belong predominantly to sterols, diterpenoids, and flavonoid glucosides, indicating a broader phytochemical diversity in native plants [39]. Conversely, the micropropagated samples contained compounds not detected in the native extracts, such as apigenin-7-O-glucuronide, kaempferol-3-O-rhamnoside, hexamethoxyflavone, and teuflin. This group is dominated by flavonoids, suggesting that in vitro conditions may promote the biosynthesis of alternative flavone and flavonol derivatives [40].

Interestingly, several compound classes showed differential representation. For example, diterpenoids such as teucrin G were found in both extracts, but salvinorin C appeared exclusively in native plants, while teuflin was restricted to micropropagated material. Similarly, flavonoid diversity was greater in the micropropagated plants, where novel apigenin derivatives were identified, whereas the native plants displayed a wider sterol profile.

In micropropagated plants, most compound classes were present at higher levels than in native material, reflecting a general stimulation of secondary metabolism under in vitro conditions. Phenolic acids such as trans-cinnamic and vanillic acid nearly doubled, suggesting an activated phenylpropanoid pathway that may enhance stress protection and antioxidant capacity. Flavonoids—notably luteolin-7-O-rutinoside, kaempferol derivatives, and diosmetin glycosides—showed the most pronounced increases, consistent with reports that tissue culture can promote flavonoid biosynthesis as part of an adaptive response to culture stress [38]. Terpenoids such as ursolic acid, tanshinone I, and riligustilide were also elevated, possibly reflecting enhanced production of defence-related metabolites. Interestingly, cycloartenol trans-ferulate and eriocitrin were unique to native plants, which may indicate that some biosynthetic branches are downregulated under in vitro conditions. Together, these findings highlight that micropropagation not only preserves but can also reprogram the phytochemical profile, potentially improving the functional value of the plant material.

In summary, while both native and micropropagated *M. croatica* share a common phytochemical backbone dominated by flavonoid glucosides and phenolic acids, each source is characterized by unique compounds that expand the overall chemical spectrum of the species. These findings emphasize the complementary value of native and in vitro cultivation systems for capturing the full metabolite diversity of *M. croatica* [19].

The ethyl acetate extracts of native and micropropagated *Micromeria croatica* revealed a considerable overlap in phytochemical composition, yet with notable differences in compound diversity between the two sources. A set of core metabolites was consistently detected in both, including albiflorin, trans-cinnamic acid, luteolin-7-O-rutinoside, teucrin G, kaempferol-O-rutinoside, salvinorin C, gallic acid monohydrate, diosmetin-7-O-glucoside, diosmetin-6-C-glucoside, kaempferol-O-rutinoside-3-O-rhamnoside, alisol F, ursolic acid, tanshinone I, and riligustilide. The presence of these compounds in both profiles indicates that they represent stable phytochemical markers of *M. croatica* regardless of cultivation system [38].

The comparative analysis of the ethyl acetate extracts of *Micromeria croatica* revealed both strong similarities and distinctive differences between native and micropropagated plants. Notably, all fourteen compounds identified in the native extract were also detected in the micropropagated material, suggesting that in vitro cultivation successfully preserves the core phytochemical profile of the species. These shared metabolites included characteristic constituents such as albiflorin, trans-cinnamic acid, luteolin-7-O-rutinoside, Teucrin G, kaempferol-O-rutinoside, salvinorin C, gallic acid monohydrate, diosmetin glucosides, alisol F, ursolic acid, tanshinone I, and riligustilide, representing a stable chemical fingerprint of the species.

However, the micropropagated extract displayed an expanded chemical diversity, with five additional metabolites absent from the native counterpart: apigenin-7-O-glucuronide, apigenin, isomaltopaeoniflorin, monohydroxyexamethoxyflavone, and dihydroxymethoxyflavanone. The presence of these additional flavonoids and glucosides highlights that micropropagation may induce or favor the biosynthesis of compounds not readily accumulated under natural growth conditions. Such differences could arise from metabolic reprogramming associated with in vitro culture, which is known to influence secondary metabolite pathways by altering hormonal balances, nutrient availability, or stress responses during plant tissue culture.

Importantly, the fact that no compounds were lost in the micropropagated extract emphasizes the reliability of tissue culture for maintaining the phytochemical integrity of *M. croatica*, while also offering a potential advantage in terms of chemical richness. From a phytochemical and biotechnological perspective, this suggests that micropropagation not only serves as a sustainable conservation strategy for rare or endemic taxa but may also enhance the repertoire of bioactive constituents, potentially broadening the applications of this species in pharmacological or nutraceutical research.

In ethyl acetate extracts, micropropagated plants consistently showed higher abundances across most compounds. Phenolic acids (trans-cinnamic, gallic acid) and flavonoids (luteolin-7-O-rutinoside, kaempferol derivatives, diosmetin glycosides) were strongly upregulated, with diosmetin-6-C-glucoside and kaempferol-O-rutinoside-3-O-rhamnoside reaching over 35%. Terpenoids, including ursolic acid, tanshinone I, alisol F, and riligustilide, also accumulated more, indicating enhanced triterpenoid and diterpenoid biosynthesis. Apigenin, a key flavone, increased six-fold, further supporting stimulation of secondary metabolism. Interestingly, monohydroxyhexamethoxyflavone and dihydroxymethoxyflavanone were detected only in micropropagated plants, expanding the chemical profile. These shifts suggest that in vitro cultivation favors phenylpropanoid and terpenoid pathways, potentially improving the bioactive potential of *M. croatica.*

In conclusion, the ethyl acetate extracts of native and micropropagated *M. croatica* demonstrate both conservation of essential phytochemicals and expansion of metabolite diversity under in vitro conditions, underscoring the value of micropropagation in both plant conservation and natural product research.

The hexane extracts of native and micropropagated *Micromeria croatica* exhibited a broad shared metabolite backbone with a limited set of differentiating constituents. A conserved panel of twelve compounds was detected in both sources: trans-cinnamic acid, luteolin-7-O-rutinoside, apigenin, teucrin G, kaempferol-O-rutinoside, salvinorin C, gallic acid monohydrate, diosmetin-7-O-glucoside, diosmetin-6-C-glucoside, isomaltopaeoniflorin, tanshinone I, and riligustilide. This overlap suggests that the principal phenolic acids, flavonoid glucosides/flavones, as well as selected diterpenoids, are consistently represented across both plant sources in the hexane fraction.

Compositional differences were modest but informative. The native extract uniquely contained monohydroxyhexamethoxyflavone, which was not detected in the micropropagated material [41]. In contrast, the micropropagated extract featured three constituents absent from the native profile: albiflorin, kaempferol-O-rutinoside-3-O-rhamnoside, and alisol F. Thus, while the shared profile is extensive, native material contributed a single additional polymethoxylated flavonoid, whereas micropropagated plants contributed two supplemental terpenoid/glucosidic constituents plus a higher-order kaempferol rutinoside derivative.

Within classes, the flavonoid domain shows partial divergence. Both sources contained luteolin-7-O-rutinoside, apigenin, and the diosmetin C-/O-glucosides; however, only the micropropagated extract included kaempferol-O-rutinoside-3-O-rhamnoside, while only the native extract included monohydroxyhexamethoxyflavone. The phenolic acid set was identical between sources (trans-cinnamic acid and gallic acid monohydrate), and the diterpenoid subset (teucrin G, salvinorin C, tanshinone I) was likewise shared, with no diterpenoids found exclusively in one source. Among other terpenoids and glucosides, isomaltopaeoniflorin was common, whereas alisol F appeared only in micropropagated plants, and albiflorin—absent from native hexane—was detected in micropropagated material [19].

Overall, the hexane extracts are dominated by a stable common core, with source-specific differences confined to a small number of flavonoid and terpenoid derivatives. Native material contributed monohydroxyhexamethoxyflavone, while micropropagated plants expanded the profile with albiflorin, alisol F, and kaempferol-O-rutinoside-3-O-rhamnoside. These presence/absence patterns refine the chemoprofile of *M. croatica* by source without altering its principal hexane-extract signature.

In the hexane extracts, micropropagated *M. croatica* displayed markedly higher abundances for most metabolites. Phenolic acids, including trans-cinnamic acid and gallic acid, were upregulated, suggesting enhanced phenylpropanoid flux. Flavonoids, such as luteolin-7-O-rutinoside, kaempferol-O-rutinoside, diosmetin glycosides, and kaempferol-O-rutinoside-3-O-rhamnoside, showed strong increases, with several exceeding 30%, indicating stimulated flavonoid biosynthesis under culture conditions. Terpenoids like tanshinone I, alisol F, and riligustilide also accumulated to higher levels, pointing to activation of terpenoid pathways. Unique compounds, including monohydroxyhexamethoxyflavone, were detected exclusively in native material, suggesting partial pathway silencing in vitro.

Overall, micropropagation favored secondary metabolism, enhancing the chemical diversity and potential bioactivity of the extracts

The results revealed that methanol was the most efficient solvent, yielding 20 identifiable metabolites in plants from native extracts and 22 from micropropagated ones. In ethyl acetate extracts, fewer compounds were identified- 19 in the extracts of plants from micropropagated versus 14 in the extracts of native ones. In the hexane extracts, even fewer compounds have been identified- 13 in extracts from native and 15 from the micropropagated ones.

The comparative analysis of methanol, ethyl acetate, and hexane extracts of native and micropropagated *Micromeria croatica* revealed a strong overlap in their phytochemical profiles, alongside distinct differences that highlight complementary metabolite expression. Across all extracts, a consistent core of phenolic acids (trans-cinnamic acid, vanillic acid, gallic acid monohydrate), flavonoid glucosides (luteolin-7-O-rutinoside, diosmetin-7-O-glucoside, diosmetin-6-C-glucoside, kaempferol-O-rutinoside, kaempferol-O-rutinoside-3-O-rhamnoside), and terpenoids (teucrin G, tanshinone I, ursolic acid, riligustilide) was maintained in both native and micropropagated plants, underscoring the stability of major biosynthetic pathways in vitro and in nature.

Differences were observed mainly in the diversity of flavonoid derivatives and terpenoids. Native plants tended to produce additional sterols and diterpenoids, such as dihydroxycholesterol, cycloartenol trans-ferulate, salvinorin C, and monohydroxyhexamethoxyflavone, which were absent from micropropagated material. Conversely, micropropagated plants exhibited a broader range of flavonoid derivatives and glucosides, including apigenin, apigenin-7-O-glucuronide, isomaltopaeoniflorin, hexamethoxyflavone, and alisol F, which were not present in native samples.

Across all three extracts, micropropagated *M. croatica* consistently exhibited higher levels of most metabolites, highlighting a general stimulation of secondary metabolism under in vitro conditions. Phenolic acids such as trans-cinnamic and vanillic acid were consistently elevated, pointing to an upregulated phenylpropanoid pathway that likely enhances antioxidant capacity and stress resilience. Flavonoids showed the most pronounced responses, with luteolin-7-O-rutinoside, kaempferol-O-rutinoside, diosmetin-7-O- and 6-C-glucosides, and kaempferol-O-rutinoside-3-O-rhamnoside all increasing significantly, several exceeding 30% abundance in ethyl acetate and hexane extracts. These changes suggest that flavonoid biosynthesis is strongly stimulated by tissue culture, aligning with previous findings linking in vitro stress to enhanced flavonoid accumulation [38]. Terpenoids such as ursolic acid, tanshinone I, riligustilide, and alisol F also accumulated at higher levels, indicating activation of triterpenoid and diterpenoid pathways. Interestingly, a few compounds, including cycloartenol trans-ferulate, eriocitrin, and monohydroxyhexamethoxyflavone, were detected only in native plants, suggesting partial suppression of specific biosynthetic branches in vitro. Together, these findings demonstrate that micropropagation not only preserves but actively reprograms the phytochemical profile, potentially improving the functional and pharmacological value of *M. croatica* extracts.

Taken together, the three solvent systems demonstrate that while both native and micropropagated *M. croatica* share a stable phytochemical backbone, each source contributes unique metabolites—native plants retaining certain sterol and diterpenoid markers, and micropropagated plants expanding the flavonoid spectrum. This complementary diversity provides a more complete picture of the species’ secondary metabolome.

### 3.1. Biological Significance of Shared Metabolites of Native and Micropropagated M. croatica

Several compounds detected in both native and micropropagated plants are well-known for their pharmacological properties (Table 5). The presence of such compounds in both native and micropropagated plants highlights the potential of tissue culture for sustainable production of bioactive metabolites.

### 3.2. Comparison with Related Lamiaceae Species

Comparative metabolomic investigations within the genus *Micromeria* reveal a highly diverse phytochemical spectrum, characterized predominantly by phenolic acids, flavonoids, terpenoids, and sterol derivatives. Abu-Reidah et al. [8] reported that *M. fruticosa* leaves are particularly rich in phenolic acids (chlorogenic, caffeic, and rosmarinic acids) and flavonoids such as luteolin, apigenin, and their glucosides. Similarly, Sarikurkcu et al. [65] highlighted *M. nervosa* extracts as sources of hydroxycinnamic acids, flavone glucosides, and flavanones, tightly linked to their antioxidant and enzyme-inhibitory activities. Al-Yousef et al. [8] identified *M. imbricata* as abundant in rosmarinic acid, catechin derivatives, and terpenoids, whereas Stavrakeva et al. [66] focused on *M. frivaldszkyana*, confirming a profile dominated by flavonoid glucosides and terpenoid metabolites, with anti-inflammatory efficacy. More recently, Yılmaz et al. [67] described *M. cymuligera* as a source of rutin, eriocitrin, and kaempferol-O-rutinoside glucosides. Comparative reviews of *Clinopodium* [68] further confirmed the taxonomic tendency toward caffeic acid derivatives, rosmarinic acid, and flavone/flavonol glucosides.

The chemical profile of *M. croatica*, based on extracts from both native and micropropagated plants, aligns with these general patterns but also reveals unique features. Across solvents (methanol, ethyl acetate, hexane), consistent markers include albiflorin, trans-cinnamic acid, gallic acid, diosmetin-7-O-glucoside, diosmetin-6-C-glucoside, luteolin-7-O-rutinoside, kaempferol-O-rutinoside-3-O-rhamnoside, and terpenoid-like compounds such as teucrin G, riligustilide, and alisol F. Micropropagated plants yielded additional metabolites (apigenin, apigenin-7-O-glucuronide, hexamethoxyflavone derivatives), suggesting metabolic plasticity induced by in vitro culture, similar to patterns noted in *M. frivaldszkyana* [66]. Moreover, the detection of salvinorin C and unusual sterol derivatives (5β-cholestane-3α,7α,12α,25-tetrol; kaempferol-O-rutinoside, and ursolic acid) differentiates *M. croatica* from other studied species, where such compounds have been rarely highlighted.

Overall, while *M. croatica* shares a core phytochemical profile with other *Micromeria* taxa—particularly phenolic acids and flavone glucosides—its distinctive terpenoid and sterol-related metabolites, along with culture-induced variations, underline both chemotaxonomic coherence and species-specific chemical diversity within the genus.

Generally, our findings demonstrate that *M. croatica* propagated via nodal explants maintains most of the phytochemical characteristics of naturally grown plants. Although minor shifts in secondary metabolite diversity were observed, particularly with methanol extracts, the core set of bioactive constituents remained largely intact. Given the conservation of major compounds with known biological activity, tissue culture-derived *M. croatica* may represent a sustainable alternative source of pharmacologically relevant metabolites.

## 4. Materials and Methods

### 4.1. Native Plant Material

The above-ground branches were harvested from a few *M. croatica* plants growing in W. Serbia on the location Beli Rzav gorge (latitude 43° 46′26″ N and longitude 19° 27′44″ E). The voucher specimen (Nº 6913) was deposited in the Herbarium collection of the Faculty of Science and Mathematics, University of Niš (HMN).

### 4.2. Establishment and Maintenance of In Vitro Cultures

Mature seeds were subjected to a multi-step sterilization protocol. First, they were immersed for 1 min in 96% ethanol containing two drops of commercial liquid detergent and then rinsed with sterile distilled water. Next, they were treated with 0.1% HgCl_2_ for 20 min and rinsed three times with sterile distilled water. This was followed by sterilization in 30% commercial sodium hypochlorite solution (equivalent to 6% active chlorine) for 25 min, after which the seeds were rinsed three more times with sterile distilled water. Subsequently, they were soaked in a solution containing 500 mg L^−1^ nystatin for 24 h, rinsed again three times, and finally washed in 0.2% KNO_3_ for 15 h.

The surface-sterilized seeds were then inoculated individually (one per glass tube) onto basal MS medium supplemented with 3% (*w*/*v*) sucrose and solidified with 0.7% (*w*/*v*) agar (Torlak, Belgrade). The pH of the medium was adjusted to 5.8 before autoclaving at 114 °C for 25 min.

Shoot cultures were obtained from aseptically germinated seedlings by cutting them into single-node explants. These were maintained in 250-mL glass jars containing 25 mL of basal medium, with ten explants per jar, sealed with polycarbonate covers. Cultures were incubated at 23 ± 3 °C under a 16-h photoperiod, with a photon flux density of 45 μmol m^−2^ s^−1^ provided by cool white, fluorescent lamps. Subcultures were routinely transferred onto fresh medium every four weeks [38,69]

### 4.3. Extracts Preparation Protocol

Air-dried plant material (1 g) was extracted with 10 mL of methanol in the presence of low-frequency ultrasound. Sonication was performed 2 × 30 min using an ultrasonic cleaning bath (Sonic, Niš, Serbia; internal dimensions: 30 × 15 × 20 cm; total nominal power: 350 W; frequency: 40 kHz). The procedure was repeated with ethyl acetate and hexane. After 0.5 h, the samples were filtered through glass funnels using Whatman No. 1 filter paper, and the filtrates were collected in 50 mL beakers. The solvents were removed under a gentle stream of nitrogen, and the solid extracts were kept frozen (255 K) until analysis. An adequate mass of solid extract was dissolved in the corresponding solvent immediately before analysis [70].

### 4.4. Liquid Chromatography—High Resolution Mass Spectrometry (LC-HRMS)

Plant extracts were prepared from both native and micropropagated plants using solvents of varying polarities. Each extract was then adjusted to a final concentration of 0.2 mg mL^−1^ prior to analysis. Chromatographic separation and mass spectrometric detection were carried out using an HPLC system coupled to an ESI-LTQ Orbitrap mass spectrometer (Thermo Fisher Scientific, San Jose, CA, USA). For each run, 5 μL of the prepared sample was injected onto a reversed-phase C18 Symmetry column (2.1 × 150.5 mm, 10 μm particle size; Waters, Dublin, Ireland). The elution was performed under isocratic conditions with a mobile phase consisting of acetonitrile and water (1:1, *v*/*v*) containing 0.1% formic acid, delivered at a constant flow of 200 μL/min.

The instrument was externally calibrated to ensure accurate mass measurements with an accuracy of ±5 ppm. Ionization was achieved in positive electrospray ionization (ESI) mode under the following settings: spray voltage 4 kV, capillary voltage 20 V, capillary temperature 275 °C, sheath gas (nitrogen) 70 arbitrary units (a.u.), auxiliary gas 20 a.u., and tube lens offset 80 V. Spectra were recorded over an m/z range of 145–900. For structural characterization, collision-induced dissociation (CID) experiments were conducted in the linear ion trap, using helium both as the collision and damping gas. Precursor ions were isolated with a window of 1 m/z, and fragmentation was induced with 15% normalized collision energy. The resolving power of the Orbitrap analyzer was adjusted to 60,000 (FWHM at m/z 400) [70].

### 4.5. Statistical Analysis

Statistical analysis was performed using Statistica 8 software (StatSoft, Tulsa, OK, USA). The level of significance was set at *p* < 0.05. The chi-square test was used to assess the differences between naturally occurring and micropropagated *M. croatica*. The data used for the Chi-square test were the presence and absence of detected compounds, and then the frequency of occurrences between native and micropropagated samples was compared.

## 5. Conclusions

This work presents the first detailed characterization of the chemical composition of both native and micropropagated *Micromeria croatica*, carried out by LC-HRMS in three solvent extracts. The comparative analysis showed that all major metabolites detected in wild-growing plants were also present in micropropagated material, demonstrating that in vitro culture preserves the characteristic phytochemical profile of the species. At the same time, the micropropagated plants produced several additional compounds, particularly in the methanol and ethyl acetate extracts, which broadened the overall chemical spectrum.

The fact that micropropagated plants maintain the same key metabolites while also introducing new ones is a significant result. It confirms that micropropagation is a reliable method for conserving the metabolic fingerprint of this endemic species, while providing a reproducible source of chemically diverse plant material. In this way, the approach offers a sustainable alternative to collecting from natural habitats and supports the long-term preservation of *M. croatica*.

By generating the first published chemical profiles of *M. croatica* extracts using high-resolution mass spectrometry, this study not only establishes a phytochemical baseline for the species but also highlights subtle metabolic shifts associated with growth conditions. Beyond advancing knowledge of a poorly characterized Balkan endemic, the findings demonstrate the value of micropropagation in conservation and sustainable utilization strategies, while opening opportunities for pharmacological, nutraceutical, and cosmetic applications.

## Figures and Tables

**Table 1:** Compounds identified by LC-HRMS in the *M. croatica* methanol extract (RT: retention time, minutes).

RT	*M. croatica* Native	Abundance of Compounds (%)	RT	*M. croatica* Micropropagated	Abundance of Compounds (%)
1.0	Albiflorin	0.52	0.98	Albiflorin	0.81
1.03	Dihydroxycholesterol	1.23	1.05	Apigenin-7-O-glucuronide	1.12
1.61	Trans-cinnamic acid	0.29	1.61	Trans-cinnamic acid	0.88
2.03	Dihydroxymethoxyflavone-glucoside	0.49	2.05	Vanillic acid	0.98
2.09	Vanillic acid	0.57	2.48	5β-cholestane-3α7α12α25-tetrol	0.68
2.51	Luteolin-7-O-rutinoside	0.82	2.51	Luteolin-7-O-rutinoside	1.00
2.48	5β-Cholestane-3α7α12α25-tetrol	0.77	3.38	Apigenin	0.74
3.67	Cycloartenol trans-ferulate	0.78	6.42	Teucrin G	0.38
6.42	Teucrin G	1.37	7.50	Kaempferol-O-rutinoside	0.52
7.50	Kaempferol-O-rutinoside	0.62	14.41	Gallic acid monohydrate	0.70
9.91	Salvinorin C	1.05	17.27	Diosmetin-7-O-glucoside	0.95
10.93	Eriocitrin	0.63	19.80	Diosmetin-6-C-glucoside	1.13
14.38	Gallic acid monohydrate	1.14	19.90	Kaempferol-O-rutinoside-3-O-rhamnoside	0.45
17.30	Diosmetin-7-O-glucoside	0.60	27.78	Kaempferol-3-O-rhamnside	3.49
19.84	Diosmetin-6-C-glucoside	0.56	28.55	Alisol F	0.37
19.90	Kaempferol-O-rutinoside-3-O-rhamnoside	1.59	32.15	Hexamethoxyflavone	0.55
23.15	Isomaltopaeoniflorin	0.57	33.93	Teuflin	2.58
28.55	Alisol F	0.36	35.94	Riligustilide	0.49
35.92	Ursolic Acid	2.93	35.99	Tanshinone I	0.41
35.99	Tanshinone I	0.56	36.04	Ursolic acid	0.31
35.99	Riligustilide	0.14			

**Table 2:** Compounds identified by LC-HRMS in the *M. croatica* ethyl acetate extracts (RT-retention time, minutes).

RT	*M. croatica* Native	Abundance of Compounds (%)	RT	*M. croatica* Micropropagated	Abundance of Compounds (%)
1.02	Albiflorin	0.18	0.98	Apigenin-7-O-glucuronide	0.31
1.62	Trans-cinnamic acid	0.80	1.07	Albiflorin	0.47
2.52	Luteolin-7-O-rutinoside	1.43	2.55	Luteolin-7-O-rutinoside	1.01
6.51	Teucrin G	0.18	1.55	Trans-cinnamic acid	0.67
7.56	Kaempferol-O-rutinoside	0.49	3.37	Apigenin	1.52
9.91	Salvinorin C	0.95	6.54	Teucrin G	0.66
14.41	Gallic acid monohydrate	0.65	7.58	Kaempferol-O-rutinoside	0.54
17.36	Diosmetin-7-O-glucoside	0.98	9.98	Salvinorin C	0.47
19.88	Diosmetin-6-C-glucoside	0.54	14.42	Gallic acid monohydrate	1.54
19.88	Kaempferol-O-rutinoside-3-O-rhamnoside	0.40	17.35	Diosmetin-7-O-glucoside	0.81
28.56	Alisol F	1.28	19.84	Diosmetin-6-C-glucoside	1.34
35.91	Ursolic acid	0.73	19.93	Kaempferol-O-rutinoside-3-O- rhamnoside	0.33
35.98	Tanshinone I	0.47	23.09	Isomaltopaeoniflorin	0.14
35.98	Riligustilide	2.16	28.49	Alisol F	0.90
			32.08	Monohydroxyexamethoxyflavone	3.32
			32.45	Dihydroxymethoxyflavanone	0.74
			35.97	Riligustilide	0.37
			35.99	Tanshinone I	0.44
			36.07	Ursolic acid	0.39

**Table 3:** Compounds identified by LC-HRMS in the *M. croatica* hexane extract (RT-retention time, minutes).

RT	*M. croatica* Native	Abundance of Compounds (%)	RT	*M. croatica* Micropropagated	Abundance of Compounds (%)
1.48	Trans-cinnamic acid	0.24	0.97	Albiflorin	0.49
2.54	Luteolin-7-O-rutinoside	0.28	1.52	Trans-cinnamic acid	1.12
3.53	Apigenin	0.48	2.57	Luteolin-7-O-rutinoside	0.67
6.46	Teucrin G	0.49	3.41	Apigenin	0.45
7.40	Kaempferol-O-rutinoside	0.43	6.50	Teucrin G	0.60
9.96	Salvinorin C	0.34	7.54	Kaempferol-O-rutinoside	0.79
14.48	Gallic acid monohydrate	0.36	9.84	Salvinorin C	0.54
17.40	Diosmetin-7-O-glucoside	1.61	14.44	Gallic acid monohydrate	0.56
19.96	Diosmetin-6-C-glucoside	0.57	17.35	Diosmetin-7-O-glucoside	0.53
23.10	Isomaltopaeoniflorin	0.53	19.85	Diosmetin-6-C-glucoside	0.72
32.02	Monohydroxyhexamethoxyflavone	3.44	19.85	Kaempferol-O-rutinoside-3-O-rhamnoside	0.55
35.97	Tanshinone I	1.19	23.17	Isomaltopaeoniflorin	1.33
35.97	Riligustilide	0.53	28.49	Alisol F	0.45
			35.95	Tanshinone I	0.35
			36.02	Riligustilide	0.52

**Table 4:** Unique compounds detected in *M. croatica* extracts by HRMS (molecular ions and corresponding fragments) (Reference literature sources with published mass spectra for the specific compound, #-ions detected as [M + Na]^+^ ion).

Number	Detected Compound	Mass to Charge Ratio (m/z), [M + H]^+^, [M + Na]^+^ #	Detected Fragmentions, m/z	Reference
1.	Albiflorin (Mr 480.5)	481.27	124.08, 214.09, 279.16, 329.14, 337.23, 359.22, 446.21, 453.12, 508.30, 595.30, 695.45, 787.41, 881.51, 940.82, 1146.48	[23]
2.	Dihydroxycholesterol (Mr 418.65)	441.22 #	148.82, 245.88, 329.14, 347.15, 508.23, 670.25, 757.32, 846.56, 857.36, 874.41, 874.41, 916.45, 1027.31, 1146.60	[24]
3.	Apigenin-7-O-glucuronide (Mr 446.36)	447.36	187.12, 214.09, 225.19, 235.20, 318.28, 391.28, 595.33, 666.91, 809.18, 940.81, 1027.26, 1146.75	[23]
4.	Trans-cinnamic acid (Mr 148.16)	171.19 #	214.09, 277.202, 299.18, 322.26, 336.27, 435.17, 595.33, 666.90, 809.15, 940.81, 1027.28, 1146.52	[25]
5.	Dihydroxymethoxyflavoneglucoside (Mr 446.4)	447.20	214.09, 271.09, 327.12, 382.20, 486.25, 597.16, 667.22, 753.30, 808.38, 853.35, 870.38, 877.36, 940.58, 1017.53, 1146.62	[26]
6.	Vanillic acid (Mr 168.14)	169.14	214.09, 299.18, 336.27, 435.17, 528.95, 598.06, 729.49, 807.97, 940.38, 1027.55, 1146.91	[25]
7.	5β-Cholestane-3α7α12α25-tetrol (Mr 711.37)	712.25	203.03, 217.05, 287.09, 362.16, 382.45, 529.02, 597.58, 712.25, 954.62, 1047.48	[24]
8.	Luteolin-7-O-rutinoside (Mr 594.52)	596.23	186.15, 205.09, 214.09, 272.14, 381.59, 444.26, 666.88, 808.87, 940.51, 1027.54, 1146.83	[27,28]
9.	Apigenin (Mr 270.24)	271.10	1947.79, 203.03, 288.12, 330.17, 529.06, 558.21, 568.14, 666.88, 808.89, 940.55, 1017.52, 1146.77	[29]
10.	Cycloartenol trans-ferulate (Mr 602.9)	604.32	182.98, 214.09, 234.13, 245.08, 345.13, 364.17, 406.22, 443.33, 529.16, 728.47, 809.14, 940.73, 1027.27, 1146.74	[30]
11.	Teucrin G (Mr 390.4)	391.28	186.95, 214.09, 264.23, 316.15, 529.20, 595.30, 772.04, 940.80, 1027.27, 1146.69	[25]
12.	Diosmetin-7-O-glucoside (Mr 462.4)	463.14	177.11, 187.04, 214.09, 245.08, 286.20, 362.16, 404.21, 529.16, 595.29, 706.29, 809.16, 940.74, 1017.43, 1146.85	[25,28]
13.	Gallic acid monohydrate (Mr 188.13)	189.13	211.09, 225.19, 264.23, 318.28, 435.17, 529.03, 597.30, 771.19, 940.50, 1017.47, 1146.76	[25,28]
14.	Salvinorin C (Mr 475.29)	476.31	179.06, 239.15, 300.20, 388.26, 432.28, 520.39, 564.36, 608.38, 666.89, 809.13, 940.78, 1027.28, 1146.46	[27]
15.	Diosmetin-6-C-glucoside (Mr 462.4)	463.12	124.09, 214.09, 275.61, 327.12, 391.29, 488.27, 530.31, 616.16, 725.33, 808.98, 941.47, 958.49, 965.46, 1025.42, 1146.57	[27,28]
16.	Eriocitrin (Mr 596.5)	597.58	203.3, 217.05, 287.09, 362.16, 382.45, 711.25, 808.17, 954.62. 1017.48, 1146.78	[24]
17.	Kaempferol-O-rutinoside-3-O-rhamnoside (Mr 740.70)	763.93 #	177.11, 187.04, 304.21, 362.16, 404.21, 528.99, 599.11, 711.25, 940.42, 1017.53, 1146.69	[28,29]
18.	Tanshinone I (Mr 276.29)	277.20	171.14, 214.09, 299.19, 322.26, 335.28, 435.17, 595.33, 666.90, 809.15, 940.81, 1027.28, 1146.52	[30,31]
19.	Kaempferol-O-rutinoside (Mr 594.52)	595.86	182.98, 234.14, 245.08, 329.14, 358.24, 488.3-27, 530.31, 770.38, 874.41, 940.59, 1146.74	[32]
20.	Kaempferol-3-O-rhamnoside (Mr 432.38)	433.17	187.13, 214.09, 264.23, 391.29, 529.20, 595.28, 666.92, 809.16, 940.80, 1027.27, 1146.68	[33]
21.	Alisol F (Mr 488.7)	489.16	187.04, 203.07, 245.08, 327.12, 362.16, 404.21, 589.21, 596.059, 706.29, 716.22, 829.31, 940.49, 1027.39, 1146.81	[23]
22.	Hexamethoxyflavone (Mr 402.39)	403.21	177.11, 214.09, 294.13, 299.18, 362.16, 529.17, 595.32, 667.57, 691.53, 809.14, 940.73, 1076.49, 1146.61	[25,28]
23.	Monohydroxyhexamethoxyflavone (Mr 252.26)	253.25	124.09, 214.09, 234.13, 299.18, 362.16, 529.31, 595.32, 667.57, 691.53, 809.14, 940.73, 1076.49, 1146.61	[25]
24.	Dihydroxymethoxyflavanone (Mr 284.27)	285.18	182.99, 214.09, 234.14, 382.41, 429.38, 447.39, 528.96, 597.41, 666.77, 808.12, 940.39, 1075.07, 1146.83	[25]
25.	Isomaltopaeoniflorin (Mr 480.46)	481.27	124.09, 214.09, 299.18, 299.18, 329.14, 446.22, 503.23, 516.26, 727.37, 809.07, 874.41, 916.45, 940.74, 1146.60	[23]
26.	Teuflin (Mr 328.4)	329.14	124.09, 214.09, 279.16, 337.24, 359.22, 446.22, 453.32, 488.27, 508.23, 596.30, 695.45, 787.41, 881.51, 940.82, 1146.48	[25,28]
27.	Riligustilide (Mr 380.48)	381.19	128.11, 203.03, 245.05, 320.11, 362.17, 488.27, 530.31, 622.20, 689.30, 738.35, 795.30, 951.45, 1027.32, 1146.78	[23]
28.	Ursolic acid (Mr 456.7)	457.24	182.99, 248.15, 272.24, 338.34, 386.28, 565.43, 666.71, 807.91, 940.42, 1017.59, 1146.75	[28,34,35,36]

**Table 5:** The biological activities of common compounds identified in native and micropropagated *M. croatica* extracts.

Common Compound	Biological Activity	References
Teucrin G	Anti-inflammatory and antimicrobial effects.	[42]
Diosmetin-7-O-glucoside	Cardiovascular protection.	[43]
Trans-cinnamic acid	Antioxidant, antimicrobial, anti-aging activity.	[44]
Vanillic acid	Antioxidant, antimicrobial and anti-inflammatory activity.	[45,46]
Apigenin	Antioxidant, antimicrobial, anti-inflammatory, antitumor, neuroprotective activity.	[47]
Riligustilide	Antioxidant, anti-inflammatory, anticancer, neuroprotective, progestogenic activity and cardiovascular protection.	[48]
Alisol F	Anti-inflammatory activity.	[49]
Kaempferol-O-rutinoside	Antioxidant, anti-inflammatory, antidiabetic, anti-cancer, hepatoprotective, renoprotective, gastroprotective, neuroprotective, cardioprotective	[50,51]
Isomaltopaeoniflorin	Alleviating pain.	[52]
Diosmetin-6-C-glucoside	Antioxidant, anti-aging, anti-inflammatory, antidiabetic activity, potential in Alzheimer’s treatment, enhance spatial memory, contribute to memory-enhancing and has anxiolytic effects.	[53]
Kaempferol-O-rutinoside-3-O-rhamnoside	Effects on the swift healing of skin injuries. Antioxidant, anti-inflammatory, cytotoxic, neuroprotective effects, and cardiovascular protection.	[54,55]
Gallic acid monohydrate	Antioxidant, anti-inflammatory, anticancer and antiviral activity.	[56]
Salvinorin C	Psychoactive activity.	[57]
Tanshinone I	Anti-inflammatory, antioxidant, and anticancer effects.	[33,58]
5β-Cholestane-3α7α12α25-tetrol	Applications in metabolic disorders	[59]
Monohydroxy hexamethoxy flavone	Anti-inflammatory activity	[60]
Luteolin-7-O-rutinoside	Antioxidant, antimicrobial, anti-inflammatory, antitumor, neuroprotective, anti-viral activity and cardiovascular protection.	[61]
Albiflorin	Anti-inflammatory, antioxidant, and neuroprotective effects.	[62]
Dihydroxymethoxy flavoneglucoside	Antioxidant, anti-inflammatory, and anticancer properties.	[63,64]

## Abbreviations

The following abbreviations are used in this manuscript:

LC-HRMS	Liquid Chromatography- High Resolution Mass Spectrometry
TIC	Total Ion Chromatogram
HMN	Herbarium collection of the Faculty of Science and Mathematics, University of Niš
HPLC	High Pressure Liquid Chromatography
ESI-LTQ	Electrospray Ionization- Linear Ion Trap
C_18_	Octadecyl silica
ESI	Electrospray Ionization
FWHM	Full Width of Half Maximum intensity
BHT	Butylated Hydroxytoluene
